# Advancing the application of systems thinking in health: a realist evaluation of a capacity building programme for district managers in Tumkur, India

**DOI:** 10.1186/1478-4505-12-42

**Published:** 2014-08-26

**Authors:** Nuggehalli Srinivas Prashanth, Bruno Marchal, Narayanan Devadasan, Guy Kegels, Bart Criel

**Affiliations:** Institute of Public Health, #250, 2 C Main, 2 C Cross, Girinagar I Phase, Bangalore, 560 085 Karnataka India; Institute of Tropical Medicine, Nationalestraat 155, 2000 Antwerp, Belgium

**Keywords:** Capacity building, District health system, Organisational commitment, Realist evaluation, Self-efficacy, Systems thinking, Programme theory

## Abstract

**Background:**

Health systems interventions, such as capacity-building of health workers, are implemented across districts in order to improve performance of healthcare organisations. However, such interventions often work in some settings and not in others. Local health systems could be visualised as complex adaptive systems that respond variously to inputs of capacity building interventions, depending on their local conditions and several individual, institutional, and environmental factors. We aim at demonstrating how the realist evaluation approach advances complex systems thinking in healthcare evaluation by applying the approach to understand organisational change within local health systems in the Tumkur district of southern India.

**Methods:**

We collected data on several input, process, and outcome measures of performance of the *talukas* (administrative sub-units of the district) and explore the interplay between the individual, institutional, and contextual factors in contributing to the outcomes using qualitative data (interview transcripts and observation notes) and quantitative measures of commitment, self-efficacy, and supervision style.

**Results:**

The *talukas* of Tumkur district responded differently to the intervention. Their responses can be explained by the interactions between several individual, institutional, and environmental factors. In a *taluka* with committed staff and a positive intention to make changes, the intervention worked through aligning with existing opportunities from the decentralisation process to improve performance. However, commitment towards the organisation was neither crucial nor sufficient. Committed staff in two other *talukas* were unable to actualise their intentions to improve organisational performance. In yet another *taluka*, the leadership was able to compensate for the lack of commitment.

**Conclusions:**

Capacity building of local health systems could work through aligning or countering existing relationships between internal (individual and organisational) and external (policy and socio-political environment) attributes of the organisation. At the design and implementation stage, intervention planners need to identify opportunities for such triggering alignments. Local health systems may differ in their internal configuration and hence capacity building programmes need to accommodate possibilities for change through different pathways. By a process of formulating and testing hypotheses, making critical comparisons, discovering empirical patterns, and monitoring their scope and extent, a realist evaluation enables a comprehensive assessment of system-wide change in health systems.

**Electronic supplementary material:**

The online version of this article (doi:10.1186/1478-4505-12-42) contains supplementary material, which is available to authorized users.

## Multilingual abstract

Please see Additional files 1 and 2 for translations of the abstract into Kannada and Hindi languages.

## Introduction

A capacity-building intervention that targets district health management teams is complex given that its implementation involves various actors with different objectives, roles, and power. Further, the setting in which it intervenes is complex since district health systems are constantly evolving in response to national policies, the local socio-political environment, and internal dynamics within the healthcare institutions [1–3]. Realist evaluation can help to make sense of the complex nature of change that is expected in a scenario such as a district level capacity-building intervention. In this paper, we aim to demonstrate how the realist evaluation approach helps in advancing complex systems thinking in healthcare evaluation. We do this by comparing the outcomes of cases which received a capacity-building intervention for health managers and explore how individual, institutional, and contextual factors interact and contribute to the observed outcomes.

### People at the core of health systems

People are at the core of health systems capacity [4]. One of the characteristics of a well-performing health system is a robust human resources management system that ensures the right conditions to achieve and maintain performance of the health workforce, which includes health managers. Health worker performance is closely related to their management capacity, but not limited to capacity alone; performance of health staff is determined by a variety of factors related to motivation, organisational dynamics and culture, and environmental factors including socio-economic and political factors [5–7]. These determinants of performance are constantly changing. From a complex adaptive systems perspective, capacity and performance could be viewed as emergent characteristics of a district health system that has many constantly self-adjusting and inter-dependent components [8].

From a realist perspective, it is not merely the implementation of programmes, but people, who change things. A programme is expected to work through providing new resources to one or more actors (agents) within this system. In response to the new resources introduced into the system by the programme, a change in the actors’ behaviour or their interactions with systemic elements could create a new way of doing things and thus result in the programme outcome. This “new way of doing things” is expected to result in better performance and hence better health services. While programmes could be designed to change behaviour of people through introducing new knowledge, skills, or ideas, we see that in complex adaptive systems, the response of the people and the systems is neither straightforward nor easily predictable.

### Building capacity and improving performance

Capacity building programmes are one of the most commonly used strategies to improve performance of health workers, especially in low- and middle-income countries [1]. However, the connection between capacity building and performance is not straightforward; capacity building is described as being multi-dimensional, spanning individual, teams, institutional, and health system dimensions. Experience from action research in several Indian settings has shown that the more we seek strengthening of systemic capacity, the more complex it seems to be and the harder it is to achieve, being rooted in organisational and the prevailing socio-cultural factors, while implementation of new skills and introduction of tools seem to be relatively less time-consuming and rooted in more technical domains [9]. In view of this multi-dimensional nature of health worker capacity (and performance), the implementation of capacity building interventions in district health systems is complex; improved performance may occur in some settings and not in others. Further, the transition from individual capacity to organisational capacity is not straightforward; several organisational factors play a role in realising the individual capacity of health managers. The disparity in results can be due to a variety of factors, including (but not limited to) the context and the actors’ perceptions of the intervention and their responses to it, their interactions with each other, their organisation, and their environment.

### Complex adaptive systems: implications for programme evaluation

The conceptualisation of district health systems as a complex adaptive system has implications for evaluating healthcare interventions. In this view, districts are sensitive to (dynamic) contextual factors as well as their initial conditions, which accounts for the often differing outcomes of the same policy or programme. On the other hand, policies or programmes may produce similar outcomes through different organisational configurations within the same district [10]. The literature on programme evaluation as well as on complex adaptive systems urges evaluation researchers and practitioners to adopt research designs that allow the consideration of unanticipated effects, adopting more flexible designs, capitalising on patterns and regularities emerging in the observations, and adopting an iterative manner of inquiry [2, 11]. Studies that embrace complex adaptive systems thinking and theory-driven methods inherently allow for these aspects as they invariably involve several cycles of observations and analysis, especially in the complex healthcare settings. In public health, programme evaluation has embraced complexity. The recently revised Medical Research Council guidance for the assessment of complex interventions, for instance, calls for a closer examination of the causal mechanisms and theory-building to contribute to developing more effective interventions, and provide insight into how findings might be transferred across settings and populations [12, 13]. However, flexible research designs for understanding change in response to interventions in a complex adaptive system may have trade-offs in terms of generating knowledge that has external validity beyond the intervention being studied. In this paper, we present a case for using realist evaluation (explained below) to explain change within complex adaptive systems such as a district health system, while broadening the transferability of results [14].

### Realist evaluation and complexity

The realist evaluation approach engages with complexity by taking an open systems approach to social systems [15]. The number of interacting agents, components, and forces that influence people and organisations in a given system is high, outcomes are sensitive to initial conditions, and thus outcomes are likely to show high variability. The realist approach to this complexity is to view reality as being stratified, with several layers of explanations to be found for the empirical observations. This provides a possibility to hypothesise and refine our explanations of why some phenomena occur [15, 16]. In the realist view, there are many possible behavioural choices that people manifest (or not) in specific conditions, which results in the outcome. An evaluation using the realist approach thus begins by seeking an explanation for why the outcome of interest occurs in some places and not in others, keeping in mind that programmes work through people and their choices. Programmes facilitate agents to make choices and interact in new ways by providing physical or symbolic resources [17].

**Figure 1:**
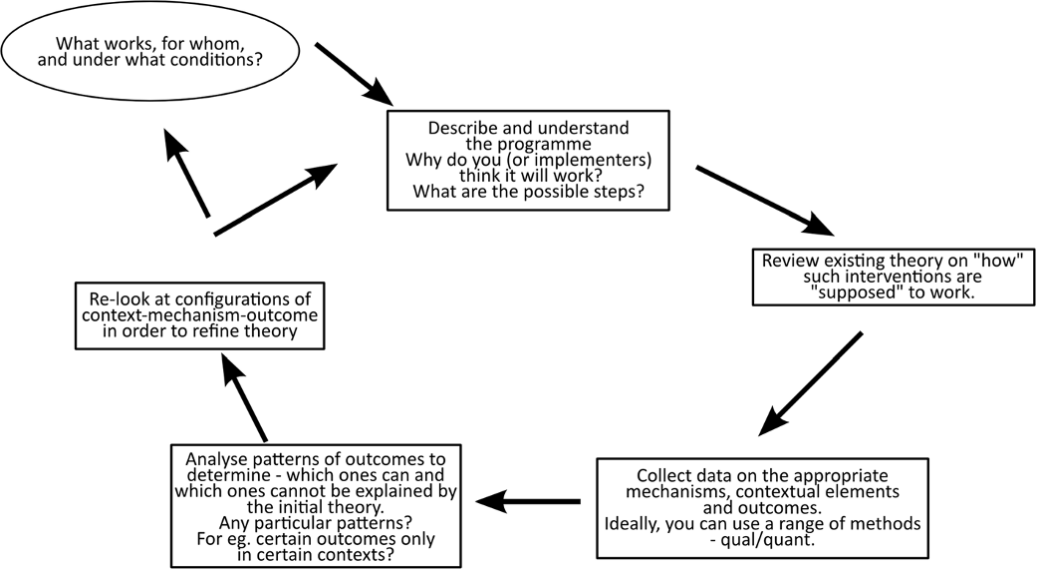
**The realist evaluation cycle showing the steps in a realist evaluation study.** Figure based on steps described by Pawson and Tilley [20].

In order to understand the relationship between intervention, context, and outcome, realists use the concept of mechanisms, which are the “… *underlying entities, processes, or* [social] *structures which operate in particular contexts to generate outcomes of interest*” [16]. In the case of complex adaptive systems, several latent mechanisms could be present within the system, which can be triggered by the intervention in the presence of specific contextual elements and result in the observed outcomes [18]. In practice, realists use the context-mechanism-outcome (CMO) relationship as a tool for empirical investigation and analysis. It allows for developing an explanatory theory of why the intervention worked for some and did not for others (Figure 1). Theoretical explanations of this kind are referred to as middle-range theories, explanations which “*…involve abstraction… but* [are] *close enough to observed data to be incorporated in propositions that permit empirical testing*” [16, 19]. It should be noted that in the literature, middle range theory and programme theory are increasingly used interchangeably. In this paper, for reasons of clarity, we will use the term programme theory.

In a realist approach, the evaluation begins with formulating a programme theory (integrating the assumptions of the programme designers and implementers with the existing wider knowledge or evidence on the topic and insight regarding the contextual factors that could affect the outcome). The programme theory is tested through empirical studies and a refined theory that explains why the intervention worked for some and not for others is the end point of the evaluation. This could be the starting point for a next study. Such cycles allow for fine-tuning of the programme theory and ultimately to accumulation of insight.

The seeking of an explanation for the patterns (or demi-regularities, which are somewhat predictable patterns or pathways of programme functioning) seen in some cases (and not in others) is the hallmark of a realist evaluation [14, 21, 22]. This addresses one of the features of complexity in social systems, wherein orderly patterns could be seen at the systems level, but often not at the individual level, due to reiterative positive and negative feedback loops among some components (and not in others) [23]. The foundations of realist evaluation within critical realism^a^, and its evolution as a scientific evaluation method are described by Pawson [14]. Its potential as an evaluation approach for complex health systems problems has gained interest over the last decade [24–28].

In this paper, we use a case study approach to explore how a capacity building intervention implemented in two different places in a district (both nested systems within the larger complex system of the district) evolved over time, using a realist evaluation, in order to understand how and why observed outcomes occurred. In line with the realist evaluation approach, cases were purposively selected to allow testing of the programme theory propositions and to improve our understanding of why programmes work for some and not for others [15]. We then use the multipolar framework to summarise how the capacity-building intervention could have led to organisational change in a district health system. The multipolar framework, inspired by Champ et al. [29], is a heuristic tool that has been used to explain organisational change in healthcare organisations in high-income settings with recent application in low- and middle-income country settings [22, 30].

### Study setting

This study is based on a capacity building intervention in Tumkur district, which is one of the 30 districts in Karnataka state in southern India; Tumkur had a population of 2.67 million in 2011 [31]. It is an average district with respect to health and development indicators; it ranked 15th in the human development index ranking of the (then) 27 districts of Karnataka in 2005 [32]. In Karnataka, poor health outcomes in maternal health have been attributed to systemic failures in managing health services and responding to critical problems service delivery [33]. Karnataka, like many other Indian states, lacks a management cadre within the health services. In Tumkur, as in all the other districts of the state, doctors with specialisation in one of the clinical specialities and several decades of experience in hospital settings are appointed as health managers of districts and sub-districts without formal or in-service management training [34–37].

The district health system in Karnataka is composed of several sub-systems called *talukas.* They are the political and administrative sub-units of the districts. In 2011, the *taluka* population in Tumkur district ranged from 168,039 in Koratagere to 598,577 in the Tumkur *taluka. Taluka* health management teams are under the charge of a *Taluka* health officer (THO). An administrative medical officer (AMO) is in charge of the hospital, while the THO has the operational responsibility for the Primary Health Centres (PHC). The THO, AMO, and other members of the *taluka* health management team hold monthly review meetings of the *taluka* in which the block programme managers^b^ and senior nursing staff participate.

**Figure 2:**
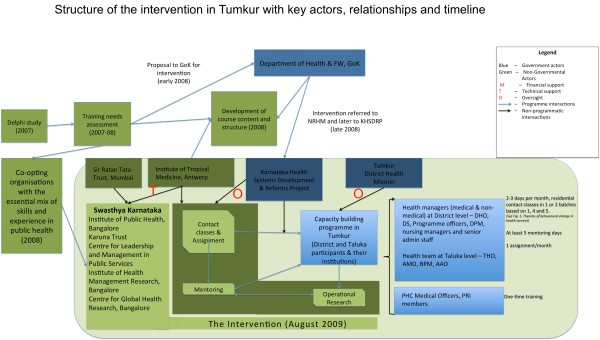
**Tumkur capacity building intervention: structure of the intervention, actors, and their roles.** Government actors are shown in blue and non-governmental actors are shown in green. M stands for financial support, *T* for technical support and *O* for oversight.

A consortium of five non-governmental organisations partnered with the state government to organise a capacity building programme for health managers of Tumkur district. The programme consisted of periodic contact classes spread over 18 months (August 2009 to January 2011), periodic mentoring visits to participants’ workplace (till December 2011), and assignments to help participants apply the knowledge and skills discussed in the classroom teaching. The aim was to bring about organisational change at the district level through improving the performance of health managers with respect to planning and supervision of health services. The intervention identified capacitated health managers as the agency through which organisational improvement could be achieved. People were seen as being at the centre of organisational change. A much shorter intervention, consisting of a one-time five-day of contact classes for all the 162 medical officers of the primary health centres of Tumkur district (all supervised by the health managers trained under the main intervention) and a facilitated discussion with *Panchayati Raj Institution* representatives (PRI), was also conducted. PRI representatives are members of the elected bodies of the local governments at village and sub-district levels. The components of the intervention and the various actors involved are shown in Figure 2. A detailed description of the intervention and its implementation has been presented elsewhere [38, 39].

In this paper, our purpose is to describe the complexity of a capacity-building intervention at the district level and illustrate the utility of the realist approach in advancing the practice of systems thinking in complex settings.

## Methods

### The realist cycle

**Figure 3:**
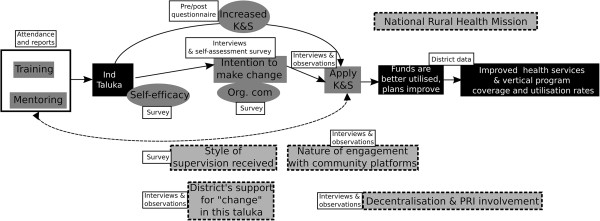
**The refined programme theory of the intervention showing possible intermediate steps between intervention inputs and expected outcomes.** Data collected for the intermediate steps are shown. Grey boxes with stippled border show contextual elements identified as having an influence on the intervention outcomes during the refining of the programme theory. Unshaded boxes indicate the source of data. Boxes shaded black indicate outcomes. Intermediate steps are shown in boxes shaded grey with no border.

A realist evaluation begins with developing the initial theory. A programme theory is best considered as an explanatory pathway, connecting the inputs of the intervention to the expected outcomes, taking into account possible contextual factors and mechanisms [40]. The refining of the programme theory, starting from the initial programme logic of the designers, to a refined programme theory incorporating insight from literature, design of the programme, and its implementation context, is explained elsewhere [41]. Our refined programme theory was aimed at explaining the differences in *taluka* outputs following the intervention, accounting for differences in the individual characteristics of the health managers, institutional factors within the two *taluka* health services and the differing environmental factors. The refined programme theory of the intervention that guided the choice of data and the analysis is shown in Figure 3.

### Case selection

**Figure 4:**
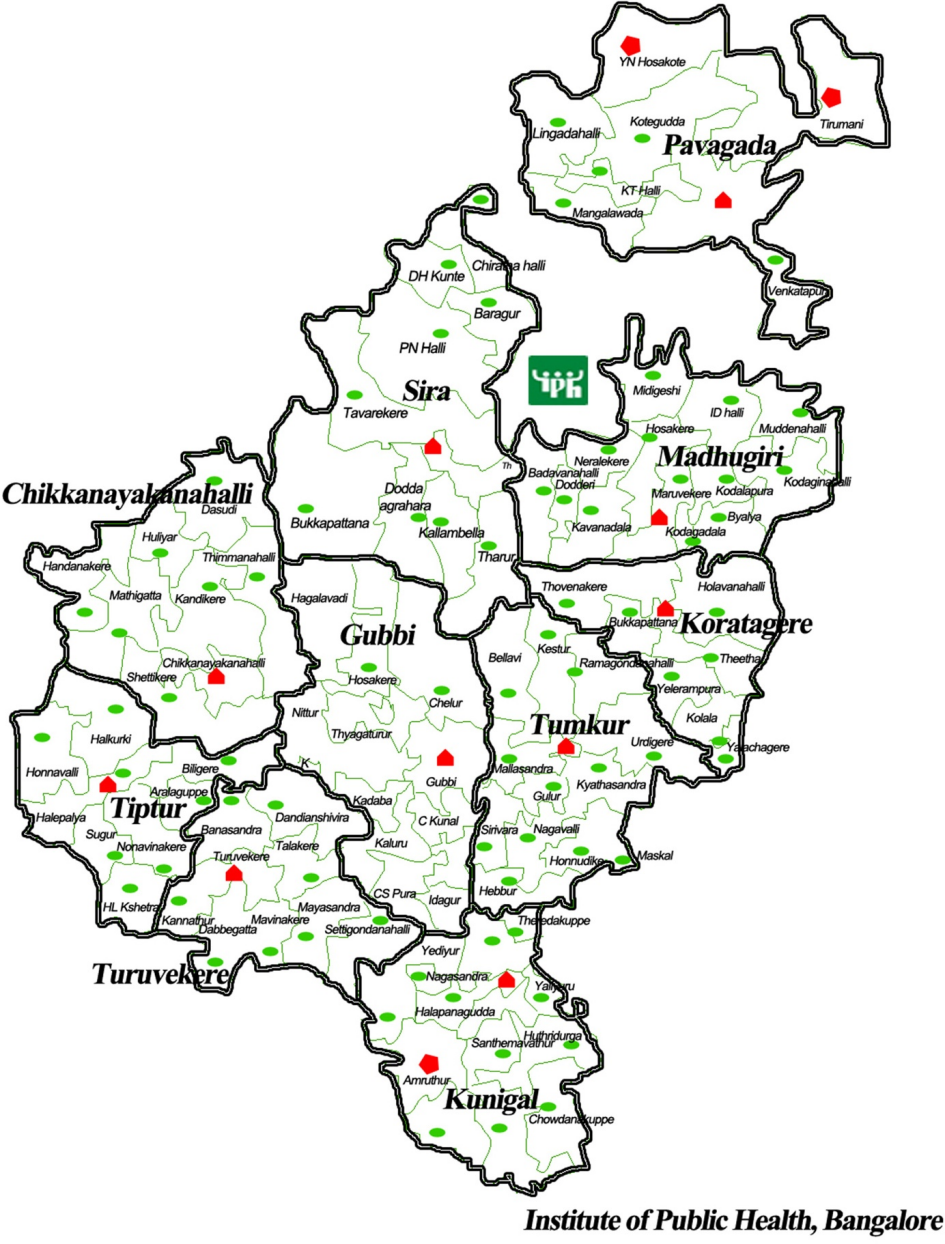
**Government health facility map of Tumkur showing the 10** ***talukas*** **, the hospitals (secondary care) and PHCs.** Green ovals show PHCs; Red polygons show secondary care facilities.

In the second step, cases were selected purposively. We assessed the performance of the 10 *talukas* of Tumkur district from 2009 to 2012, focusing on performance aspects that could be logically connected to the capacity building intervention (using the programme theory of the intervention as a guide). We scanned *taluka* performance with a focus on those showing least and most improvement; we chose one positive and one negative outlier (contrasting case selection) for the analysis presented in this paper. Figure 4 shows the *talukas* of Tumkur, including the *taluka* hospital and the PHCs.

### Data collection

In realist evaluation, the choice of data to be collected is guided by the programme theory. First, we collected data on the intensity of the programme implementation: participation in classroom activities, frequency of mentoring visits, and retention of mentoring interest. The mentors seem to have preferred *talukas* based on their own assessment of interest shown for mentoring by the *taluka* team. Hence, retention of mentor interest has been chosen as a proxy for the *taluka*’s commitment towards the vision for change as articulated by the intervention. It was assessed on the basis of frequency of mentoring visits and observation notes of the mentors, and scored into high, moderate, and low. Second, we assessed intermediate outputs (self-efficacy, organisational commitment, style of supervision, and expression of intention of *taluka* managers to make changes) using data from a survey of health managers in Tumkur.

Organisational commitment along with self-efficacy has been described as being crucial to performance and is considered as a key mechanism explaining human agency in various settings [42, 43]. The three-component construct of organisational commitment by Meyer and Allen describes the nature of commitment of people to their organisations along three dimensions: affective commitment (emotional attachment to the organisation; a feeling of belongingness), normative commitment (a feeling of being obliged to the organisation), and continuance commitment (a feeling of being in the organisation because of a lack of alternatives) [44]; the three different dimensions of commitment co-occur. Self-efficacy was measured using a 10-item scale based on the Bandura scale [45] and degree of supportive nature of supervision was measured using a Likert scale questionnaire adapted from a tool by Oldham and Cummings and the Michigan Organizational Assessment Package [46, 47]. The tools used have been described earlier and published elsewhere [39].

To assess the distal outputs of the intervention, we collected annualised data on budget utilisation, provision of 24/7 PHC services, coverage rates of institutional delivery, delivery by caesarean section (CS), completion of three antenatal care visits, and immunisation. We also assessed changes in infant mortality rate and stillbirth rate from 2008 to 2012. Stillbirths and infant mortality reported in all the facilities of the *taluka* were used to calculate the rates. These quantitative data were supplemented with qualitative data collected through interviews with health managers and observations. In-depth interviews were conducted with 21 health managers of Tumkur who participated in the intervention, their superiors at state level (n = 2), and their subordinates (PHC health staff and co-workers; n = 4). Participant observation of monthly and annual review meetings at the *taluka* and district level was carried out to understand the organisational dynamics and the differences in interpretation and implementation of state policy.

### Analysis

All interviews were transcribed and entered into NVivo 10 (QSR International Ltd., Australia), together with the observation notes. During the analysis, we used the CMO as a heuristic tool (Table 1). These hypothetical CMO frames were based on the refined programme theory of the intervention, as described elsewhere [41]. Initial codes reflected the programme theory elements of intervention, actors, context, mechanism, and outcomes, and new codes emerged. The quantitative data, including measurements of organisational commitment, self-efficacy, and style of supervision provided were integrated into the analysis and this helped in triangulating emerging findings. In this way, each case was analysed.

**Table 1:** **Identifying context-mechanism-outcome frames based on the programme theory of the intervention**

Programme inputs (IPT) and how they were supposed to work	Key assumptions identified during the refining of IPT	Supporting theory	Key contextual factor (C)	Outcome of interest (O)	Plausible mechanism (M)
Contact classes work through improving knowledge and/or skills, resulting in improved performance	An attitudinal change among the participants is needed to achieve the desired results	Outcomes of training programmes accrue through four hierarchical levels: reaction (to training programme), learning, behaviour, and impact [48]	Team dynamics affect the individual’s intention for positive change	Intention to make positive changes	Motivation of the participant towards positive organisational change – a “can-do” attitude
			Socio-political environment in the *taluka*/district		
Mentoring participants at workplace facilitates application of knowledge and skills	Targeting individuals will produce impact through teams	Workplace environment in healthcare organisations has been identified as an important element explaining application of learning from training programmes [49]	Nature of supervision and district’s openness to “allow” change	Identify/seek opportunities to make positive change in the organisation’s performance	Nature of commitment to organisation
			Decentralised action plans and decision-making at district and lower levels. State and higher levels’ openness to change proposals	Improved annual action plans – better situation analysis, problem identification, allocation and utilisation of resources	Self-efficacy
A capacitated health manager can become an agent of positive organisational change	Capacity leads to performance	High commitment management literature shows the potential for change by committed staff in settings where resources could be mobilised [50]	Change proposals by districts are in line with state (or central) vision and address local needs (allocation and strategic alignment with external environment per Champ et al.’s conceptual framework) [29]	*Taluka* and district plans improve. They identify more needs, mobilise more resources from state, and utilise them better	Claiming and utilising decision spaces; organisational commitment and self-efficacy in negotiating with superiors and community leaders
	The programme could benefit from alignment with existing policy initiatives				

We then compared the two *talukas* to further test whether the refined programme theory explained the differences in the outcomes. We supplemented these two contrasting case studies with demi-regularities from comparable settings in the other *talukas*. We focused on the internal dynamics within the *taluka* teams (micro-context) and the interaction of these teams with the immediate *taluka* environment (meso-context) and the larger policy environment at the district, state, and above (macro-context). We also described the organisational configurations of the two cases using the multipolar framework.

## Results

### Outcomes

**Figure 5:**
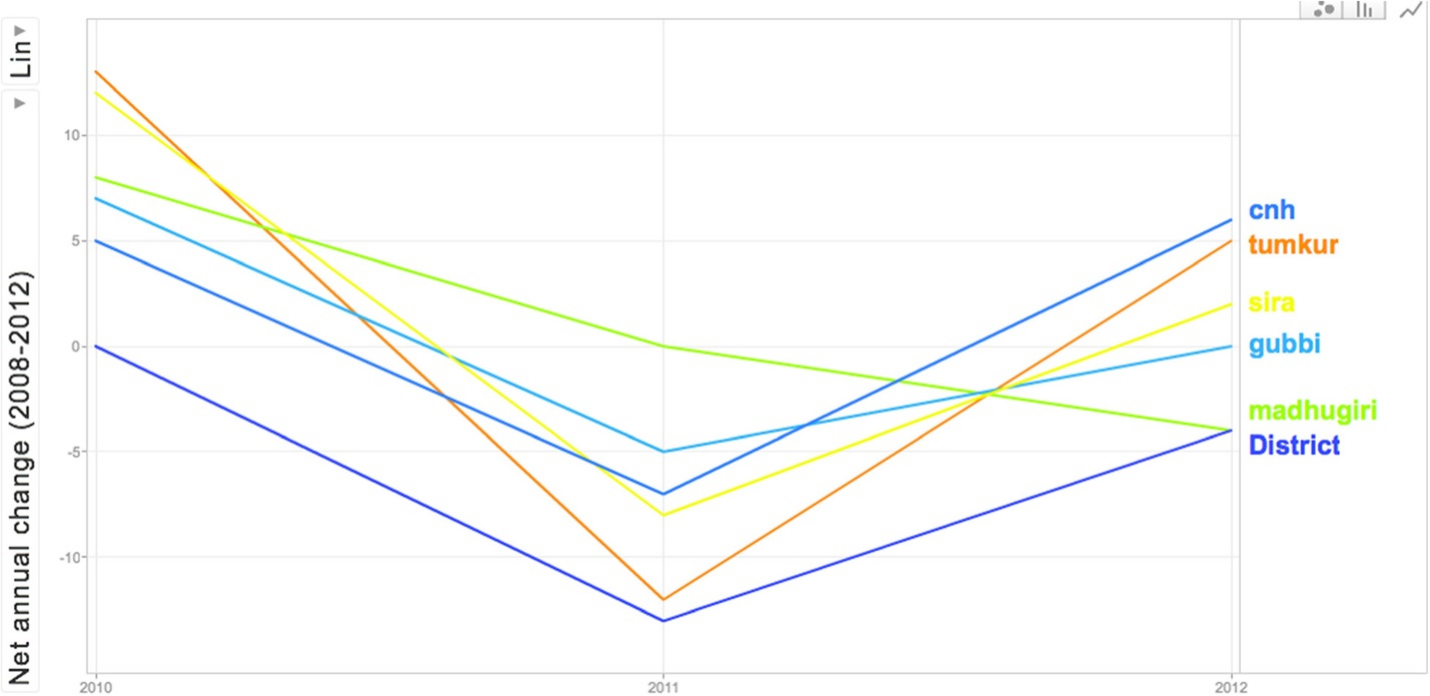
**Annual change in utilization rate of selected** ***talukas*** **of Tumkur district from 2010 to 2012.** The net change (from the previous year) in the aggregate budget utilization rates of all facilities in the *talukas* are shown for CN Halli, Tumkur, Sira, Gubbi, and Madhugiri *talukas*. The District figures are for utilization rates of budget allocated for disease control programmes and other functions managed at the district level.

The responses of the *talukas* to the intervention varied, as shown in Table 2. The aggregated budget utilisation rate for Tumkur district^c^ increased marginally, from 83% in 2009 to 85% in 2012. However, this conceals a variety of responses at *taluka* level. In Figure 5, the net annual change in utilisation (the net change in the proportion of available funds timely spent between two years) from 2010 to 2012, is shown. While some *talukas*, like Pavagada, improved their utilisation rate, others, like Madhugiri, reduced their spending rates. Yet others, like Turuvekere, showed wide changes from one year to another, while net change from 2012 to 2009 was only marginal.

**Table 2:** **Assessment of exposure to intervention, key intermediate mechanisms (commitment and efficacy), and outcomes of the 10** ***talukas*** **of Tumkur**

	***Classroom participation*** ^***1***^	Mentoring ^2^	Retention of mentoring ^3^	***Organizational commitment*** ^***4***^	Self-efficacy ^5^	***Supportive supervision*** ^***6***^	Intention to change ^7^	Stability of team ^8^	Net change in budget utilisation ^9^	Net change in CS rate ^10^	Net change in stillbirth rate ^11^	Development index ^12^
Gubbi	0.7	0.7	High	AC 2.66	68	2.5	50	Moderate	2	1	-16	0.95
				NC 2.47								
				CC 2.42								
Tumkur	0.7	0.7	Moderate	AC 2.85	68	2.6	75	Low	6	1.5	-8	1.21
				NC 2.46								
				CC 2.69								
CN Halli	0.6	0.5	Moderate	AC 2.75	70	2.2	100	High	4	0.1	0	1.02
				NC 2.29								
				CC 2.71								
Turuvekere	0.6	0.4	Low	AC 2.81	68	2.4	83	High	5	5.8	-4	1.06
				NC 2.80								
				CC 2.47								
Tiptur	0.5	0.5	Moderate	AC 2.25	86	2.5	75	Low	-4	12.6	-1	1.25
				NC 2.33								
				CC 3.17								
Koratagere	0.4	0.5	Low	AC 2.87	71	2.3	20	Moderate	3	1.8	-3	0.89
				NC 2.73								
				CC 3.07								
Madhugiri	0.5	0.5	Low	AC 2.50	83	2.4	40	High	4	1.3	-1	0.82
				NC 2.03								
				CC 2.50								
Pavagada	0.6	0.5	Moderate	AC 2.50	79	2.3	0	High	6	0	1	0.78
				NC 2.05								
				CC 2.28								
Kunigal	0.6	0.5	High	AC 2.12	83	2.2	75	Moderate	2	4.9	-4	0.96
				NC 2.59								
				CC 2.83								
Sira	0.7	0.9	High	AC 1.80	68	2.2	100	Moderate	6	8.3	2	0.81
				NC 2.00								
				CC 2.67								

^1^Average of degree of classroom participation of all participants from a *taluka*, based on assessment of attendance and classroom activity (assessed by observation notes) expressed on a scale of 0 to 1.

^2^Average of degree of mentoring received based on attendance of participants at mentoring sessions (0 to 1.0).

^3^Qualitative assessment of the *taluka*’s ability to retain interest of the mentor expressed as high, moderate, and low.

^4^Three dimensions of organisational commitment: Affective commitment (AC), normative commitment (NC), and continuance commitment (CC)*.* Individual commitment measures for each of these three dimensions were computed and the averages of these were calculated by *taluka*. Commitment scores are on a scale of 0 to 5.

^5^Self-efficacy scores expressed on a scale of 0 to 100.

^6^Style of supervision largely assessing supportive nature of supervision (1 to 5; 1 being most supportive and 5 being most authoritative).

^7^Percentage of ever-trained members in the *taluka*, who expressed intention to make changes based on the capacity building programme.

^8^Stability of team assessed based on turnover of health managers in the *taluka* team from 2009 to 2013 expressed as high, moderate, and low. High indicates stable teams (low turnover).

^9^The net change in percentage budget utilization from 2009 to 2012. Budget utilisation for each of the PHCs in the *taluka* was obtained.

^10^The net change in proportion of caesarean sections (CS) among total deliveries from 2009 to 2012. CS at *taluka* hospitals is at present very low and efforts are on to improve emergency obstetric care at *taluka* hospitals through ensuring facilities to perform CS.

^11^The net change in stillbirth rate (of the total live births in the *taluka*) from 2009 to 2012. Negative change indicates a fall in stillbirth rate.

^12^The socio-economic development index for the *taluka*. Scores less than 1 are considered very poor and such *talukas* have been designated “backward” [51].

The tools for measuring organizational commitment, self-efficacy, and supportive supervision notes on their validity in Indian settings are discussed elsewhere [39].

**Figure 6:**
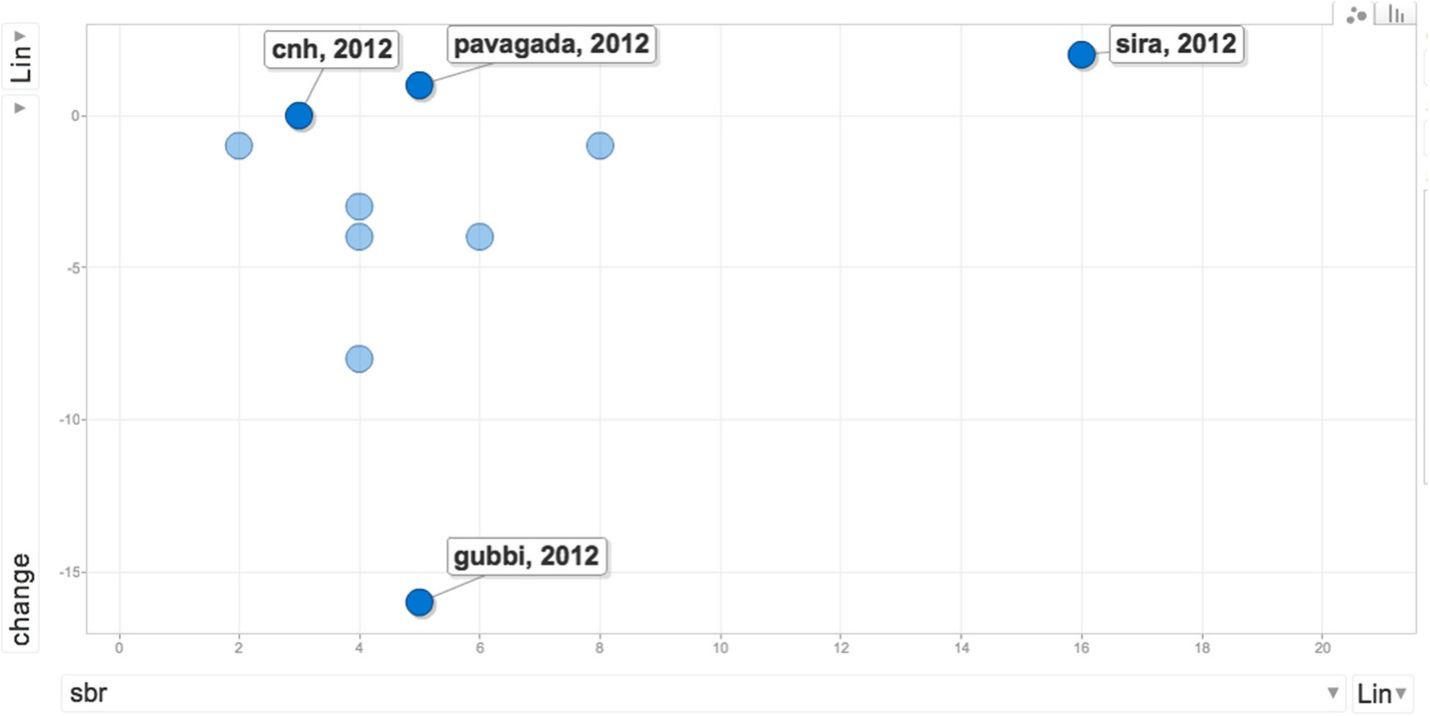
**Stillbirth rates in 2012 by** ***taluka*** **shown against net change in this indicator from 2009 to 2012.** Gubbi, Sira, Pavagada, and CN Halli stillbirth rates are labelled.

In Figure 6, the stillbirth rate in 2012 is plotted by *taluka*, against net change in stillbirth rate from 2009 to 2012. We use the net change in stillbirth rates as a proxy indicator of performance. Stillbirth was chosen because of the emphasis in the intervention on using planning (through good annual situation analyses and problem identification) and supportive supervision in improving maternal and child health outcomes. Such variability could result from several factors, including existing reform processes that promote institutional deliveries, and improvements in the functioning of the health services (including the capacity building intervention). Besides such interventions, which influence all *talukas* to the same degree, context-specific socio-political factors and organisational factors, which are of interest in our evaluation lie, within the *taluka* health services and could influence performance. We shall use the variability in the *taluka* level outcomes to purposively choose *talukas* and examine if the hypothesised explanations from the refined programme theory could explain these differences.

In Table 2, the various individual, team, and institutional factors that we assessed based on the programme theory are shown. The factors chosen are a mix of individual and organisational contextual factors (intervention exposure, socio-economic development index of *taluka*, mentoring interest and supervision received, and team stability), mechanisms of human agency at the individual level (intention to change, organisational commitment, and self-efficacy), and proxy measures of outcomes logically related to improvements in the *talukas* expected from the intervention as well as more distant *taluka* outcomes determined by several other factors. The *talukas* varied in their participation in classroom and mentoring activities, in view of transfer in and out of health managers in the *taluka* or absenteeism (either by choice or due to priority work at the *taluka*). Higher participation in the intervention did not always result in an intention to make changes at the workplace (e.g., Gubbi and Tumkur with highest participation and only moderate expressions of intention for positive change); nor did expressions of such intentions always result in improved outcomes (e.g., CN Halli with a 100% of the team expressing intent but showing negligible change over the three years in the outcomes).

We purposively present the summary of the analysis of two contrasting cases – Gubbi and CN Halli – among the 10 *talukas* to illustrate how the CMO lens derived from our refined programme theory can be used to understand and explain how the outcomes in these cases could have come about and what could be the possible contribution of the intervention in these outcomes. We present the summary of the analysis of the empirical data in the form of observed outcome (O) in relation to mechanisms (M) and contextual conditions (C).

### Gubbi

**Figure 7:**
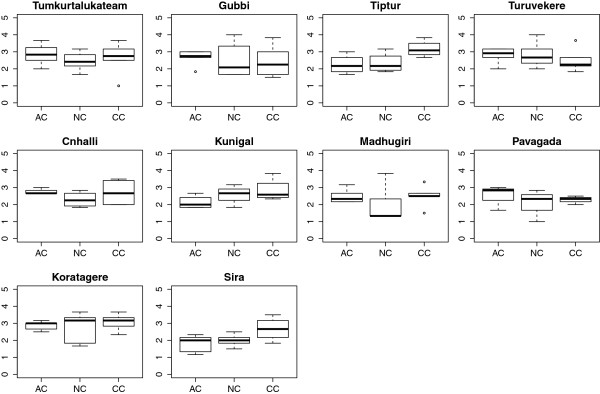
**Boxplots of three dimensions of organisational commitment in the 10** ***talukas*** **of Tumkur district.** The three dimensions of commitment are based on Meyer and Allen [44]. AC is affective commitment, NC is normative commitment, and CC is continuance commitment. Individual commitment measures for health managers were computed separately for AC, NC, and CC. For each *taluka*, box plots of the scores for each of these were plotted.

Gubbi’s stillbirth rate decreased the most among all the *talukas* in Tumkur; the improvements in proportion of CS performed and budget utilisation were modest (Table 2). Health managers from Gubbi participated actively in the intervention and retained the mentors’ interest. They showed relatively higher affective commitment than many other *talukas* (Figure 7). Only half of the health managers expressed an intention to make changes.

From the interviews and observations at Gubbi, the main theme emerging was commitment. The interest shown by the THO and the AMO towards improving services is evident from the interviews. The THO was given temporary charge of heading the team while simultaneously being the medical officer of a nearby PHC. Yet, he felt that he could mobilise greater support to improve services in the *taluka* by motivating like-minded people. He felt that being a health manager is an opportunity to bring about changes.

“*In my taluka for example, I think we can make big change. It is not that everybody in my taluka wants to make changes. Only one-third of them are motivated to make changes. And that is enough. I think I can make a lot of improvement by motivating these people.*”

*Taluka* health manager from Gubbi (g1)

Such positive assessment of motivation of PHC staff as a strategy towards improving services was not shared widely in the other *talukas*.

Both the AMO and the THO saw the intervention as an opportunity to benefit from the recent efforts to decentralise the preparation of action plans to *taluka* and PHC level. They felt that the decentralisation of planning under the National Rural Health Mission (NRHM)^d^ was an opportunity to address specific problems at the PHCs.

“*More resources mean more opportunities to make change. If they slowly give more and more power to us at taluka level, we can make many more improvements. Right now, very little is possible at taluka level.*”

*Taluka* health manager from Gubbi (g2)

“*NRHM has given block programme managers. This will improve plan preparation and monitoring. They are young and enthusiastic, but they need to some guidance and I think I can provide that.*”

*Taluka* health manager from Gubbi (g1)

This general pattern of commitment at Gubbi is also seen in the Tumkur *taluka*, with a relatively high affective commitment, albeit with a higher turnover of staff.

The Gubbi pattern could be summarised as follows: in a decentralised taluka health system, committed health managers can make use of their increased management capacity to identify opportunities for improving their health services performance.

### Chikkanayakanahalli (CN Halli)

While Gubbi is situated close to the district headquarter town of Tumkur, CN Halli is further away, but with a similar level of socio-economic development (Table 2). CN Halli showed hardly any change in most outcomes, in spite of a high intention among the health managers to make improvements in the *taluka*. CN Halli also had lower turnover rates of *taluka* level health managers. The level of affective commitment was comparable to that at Gubbi, but continuance commitment was relatively higher.

CN Halli is amongst the most remote *talukas*. With a limited private sector, it is not a favoured choice of posting for doctors. For several months, the function of THO and AMO was taken up by the same person. The *taluka* level staff showed commitment towards the services and took pride in working in a remote *taluka* with very limited human resources. However, during discussions about decentralised planning expressed by this *taluka*’s health managers, the dominant theme was frustration.

“*What PIP*^*e*^*? What decentralisation? I sent so many requirements for staff and proposals for improvement. Only thing I got is more work, less staff and zero solutions. On one hand, I have to answer the local ZP*^*f*^*members’ complaints and on the other hand, I have to just keep implementing plans and schemes coming from above. Nothing can be done without more staff.*”

Health manager from CN Halli (cnh1)

While the decentralised planning brought about by NRHM was perceived as an opportunity in Gubbi, in CN Halli the respondents expressed frustration. This was also evident in several meetings at the *taluka* level, where a lack of power to make changes at the *taluka* and district level, for instance in recruitment of human resources and purchase of critical equipment, was often raised.

“*NRHM has just brought more and more responsibilities, but no powers. For everything, we have to wait for a visit from the secretary or commissioner. More money means more work and more statements of expenditure and paperwork.*”

PHC health worker from CN Halli *taluka* at a review meeting (cnh2)

Similar frustrations about increased paperwork and responsibilities were found in the thematic analysis of interviews and observation notes from Pavagada, another poorly staffed, and the most remote *taluka* in Tumkur.

“*The increased money with NRHM is good. But it’s not merely money. We need committed people who can stay in such a remote area. I am from this area and I live and work here. People who come here hardly stay beyond a few months. They either get frustrated or seek transfers.*”

–Health manager from Pavagada (P1)

The recent reforms towards giving greater powers to the elected representatives were seen as a threat to their functioning. The *taluka* health staff felt that channelling the frustrations of the PHC staff upwards was their role much more than managing conflicts and frustrations or building amicable relationships with the elected representatives.

“*Nothing much can be done without giving powers at taluka level and PHCs. I cannot even appoint a Group D staff. Where is decentralisation in this?*”

a PHC staff from CN Halli *taluka*

“*What more can I do? I communicate promptly to my superior all the problems and I am still waiting for the solutions. In the* [capacity building] *programme they are saying, find local solutions. With so little staff, how much local solutions can I find? People just don’t want to work here. I handle two responsibilities at the same time…*”

*–* Health manager from CN Halli (cnh1)

The pattern of CN Halli is also seen at the Pavagada *taluka*, which is also severely under-staffed, with a small group of health managers with comparatively lower levels of affective commitment. The improvements of the Pavagada *taluka* were poor, in contrast to the Sira *taluka*, which is also geographically remote and socio-economically poor, yet showing a remarkable vision in the *taluka* team to operationalize emergency obstetric facilities in the hospital, a dire need in this remote region. The Sira *taluka*, unlike Pavagada and CN Halli, was much more dominated by a continuance commitment rather than affective commitment.

“*We felt that we have to do it. So many mothers were just being referred to Tumkur. The delivery load is high and for several months, we had only one obstetrician, but somehow we managed. I know how the pressure is at the district hospital, so having LSCS facility at Sira decreases the burden at the district hospital. It’s not easy, but somehow it is happening.*”

Sira health manager (s1)

The pattern of CN Halli could be summarised as follows: Health managers working in poorly resourced *talukas*, in spite of their improved management capacities and intentions to make change, get frustrated by the lack of facilitating action from above.

## Discussion

Health system interventions need to take into account the subunits of the local health system in which they intervene. In this case, each *taluka* can be conceived as a sub-system with a particular organisational context but a similar macro-context, exposed to the same intervention. In such cases, the realist evaluation approach helps to formulate specific CMO-based propositions that can be tested through comparing contrasting cases. This allows for building explanations on how organisational change occurred in some settings and not in others. The process of testing and refining the CMOs allows for an understanding of the conditions through which such interventions could work in a complex local health system.

### Explaining change: contribution of the intervention

While the training programme (the intervention) included all health managers in the district, their actual participation was variable. This depended on several factors at the level of the participant (their interest and motivation), distance between the *taluka* and the district headquarters, the staff turnover rate, and the responsiveness of the implementers to the *taluka* teams. Many of these factors are related to each other, sometimes counterintuitively. For example, remote *talukas* like CN Halli and Pavagada had a relatively low turnover, while more sought-after *talukas* like Tiptur and Tumkur *taluka* had a higher turnover. Capacity building interventions that seek to strengthen local health systems ought to take into account such existing variations within the sub-systems at the design stage.

Health system strengthening interventions seek to strengthen core systemic functions of the local health system. The capacity building intervention sought to improve performance through improving planning and supervision. The contribution of such improvement (if any) ought to be framed against several other activities at the PHC, *taluka*, and district levels. For example, the provision of secondary level obstetric care at the *taluka* hospital includes developing the capacity of the facility to conduct CSs; this has been the policy focus in Karnataka for several years. In addition to the state government’s pressure to implement this, health managers also face the pressure of the community and local elected representatives to operationalize CS facilities at *taluka* hospitals. However, in spite of favourable environmental conditions at the *taluka* level, effectively ensuring this requires a strong managerial vision and leadership; this was observed only in some *talukas*. This illustrates that, in a district health system influenced by several policies and environmental factors, it may be difficult to disentangle the contribution of the intervention to the observed outcomes. However, by choosing intermediate and distal outcomes at various levels (individual and institutional) that are most sensitive to the intervention inputs, it is possible to identify *talukas* where the intervention could have contributed to the outcome by seeking alignments with existing conditions and the characteristics of the people and teams in these *talukas*.

Capacity-building interventions could work through identifying such existing alignments between local actors’ needs, policy, and practice, and by strengthening conditions for the same. As the CN Halli case shows, in spite of favourable policy, community pressure, and a committed team at CN Halli, the frustrations of health managers resulting from previous negative experience with decentralised planning altered their choices and collective agenda-setting against actualising CSs in their hospital. In contrast, health managers of Sira *taluka* showed relatively low levels of affective commitment and self-efficacy, but frustration was low. With the participation of elected representatives and through effective leadership by the AMO, the CS facility was organised. Thus, in a *taluka* considered to be poorer than CN Halli in terms of socio-economic development indicators, the proportion of deliveries conducted by CS increased by 8.3% between 2009 and 2012. Further thematic analysis of *talukas* that resemble some of the characteristics of our cases (such as the case of Pavagada discussed under the CN Halli case summary above) or are contrasting with our cases in some respects, could strengthen our findings and allow validation of these findings in future studies in similar settings.

### From individual change to systemic change

Although the capacity building intervention was implemented at the district level across all *talukas*, the exposure to the programme, the response to the intervention (attitudes towards change and intentions), the internal individual and organisational dynamics, and the outcomes, varied. These factors determine why programmes implemented at the district level may or may not achieve their expected outcomes, especially in those healthcare institutions where the conditions necessary for such a change do not exist. However, despite this potential for variation, formulating hypotheses in the form of CMO propositions and testing these empirically can help identify patterns of response to intervention. The resulting CMO configurations can then be refined further by testing them in other cases of the district to arrive at an explanatory theory that elucidates what worked, for whom, and under what conditions*.*

**Figure 8:**
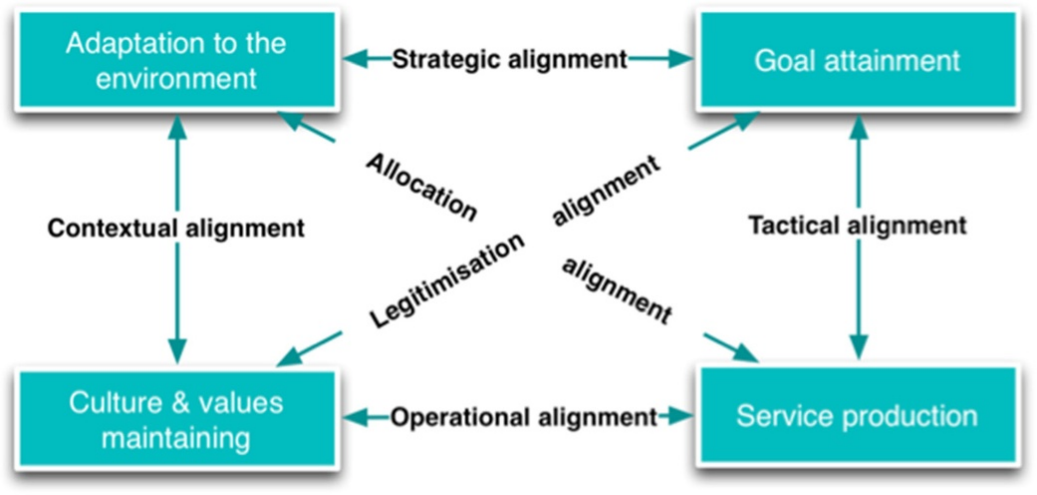
**The multipolar performance assessment framework based on Sicotte et al.** [29]**.** The framework consists four poles and six alignments.

Capacity building interventions work through people and the choices they make. Many individual attributes, such as organisational commitment and self-efficacy, have been reported as mechanisms that explain human agency [42, 44, 52]. However, the *taluka* health system is more than a group of individuals with varying commitment or efficacy measures. The change in the organisation comes about through the interaction among these participants, governed by rules and norms within their organisation (the organisational culture and their activities that result in the organisational outputs), and the interaction between the organisation as a whole with the external environment. These relationships between the internal and external components of the organisation have been brought together in the multipolar framework for assessing performance of healthcare organisations, shown in Figure 8. The multipolar framework is based on Parsons’ theory of social action and inspired by the work of Champ et al. [29, 30, 53].

**Figure 9:**
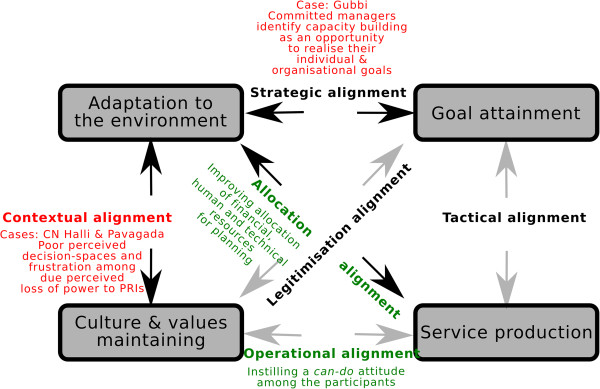
**The alignments that the intervention sought to influence to improve performance are shown in green.** The alignments that explain the responses of the cases are shown in red.

The observed changes in the *talukas* could be seen as having occurred through shifting or triggering of any of the six alignments in the multipolar framework. The *taluka* management team is responsible for managing not only the four core functions (the boxes in Figure 8), but also the alignments (the arrows in Figure 9) between the functions. The local configuration of these functions, and the management team’s response to tensions between these functions explains the variation in the outcomes of the capacity building programme.

The capacity building intervention sought to alter the outputs (service production) through increasing knowledge and skills to develop annual action plans and supervision functions. An analysis of the programme theory of the intervention indicates that the designers of the intervention sought to bring about these changes through instilling a *can-do* attitude among the health managers. This could be seen as trying to strengthen the *allocation* and *operational* alignments in the multipolar framework (Figure 8 and Figure 9). However, in the context of a health system that is undergoing decentralisation to the district levels, and where participation of elected representatives within formal structures of the health service is being increasingly pushed for by the national and state policy, the contextual alignment could dominate in some *talukas*, as was the case in CN Halli. However, a committed leadership at the *taluka* level could counter the negative perceptions of participation of elected representatives prevailing within the health service. In such cases (as in Gubbi; see Figure 9), the legitimisation and strategic alignments could be triggered where the capacity building programme was seen as an opportunity to translate existing commitment towards the organisation into an improvement in its performance. The overall performance of the *taluka* is the result of how the alignments between the four poles are perceived locally and managed. The capacity building programme thus acts upon the *taluka* performance through imparting skills and vision to managers, who then balance or counter the emerging alignments. However, it must be emphasised that in our study, the insights from the evaluation were not periodically fed back into the system to enable the local actors (implementers of the intervention and the recipient health managers) to benefit from or reflect on these. Realist evaluation could also be used as an entry-point for action research on local change, wherein the CMO frames being considered or the refined programme theory could be shared periodically with local actors. Furthermore, such discussions and sharing with local actors could be further used to refine or validate the middle-range theory emerging from the evaluation.

### Realist evaluation and systems thinking

Realist evaluation adopts a generative perspective on causality, according to which change occurs as a result of the interaction between actors within a specific context [54–56]. A programme theory that is constructed along these lines can be tested in a reiterative manner and allows for comparison across cases. The resulting insight, in the form of a refined programme theory, informs policymakers, managers, and funders on what works, for whom, in which conditions, and how. A realist evaluation of an intervention provides an explanatory theory on why the intervention worked for some and not for others through a process of adjudication between rival explanations. By employing the classical apparatus of the scientific method – “*formulating hypotheses, making critical comparisons, discovering empirical patterns, and monitoring their scope and extent*” – realist evaluation enables a comprehensive assessment of system-wide change [15].

### Limitations

The output of a realist evaluation is a programme theory or a middle-range theory (not a universal overarching theory), which provides a plausible explanation for the outcomes of the intervention; it cannot make predictive statements about the intervention. However, such middle-range theories form the basis for improving our understanding of complex interventions and help in improving design and implementation of such programmes in future.

In this paper, outliers have been purposively selected based on outcomes that are logically connected to the intervention inputs. The explanation that we provide suffers from a possible confirmation bias. Ideally, a full realist evaluation needs to refine the middle-range theory through several iterations of cases selected based on diversity of outcomes. This will strengthen the explanatory power of the middle-range theory.

In an open systems world, there is no end to the explanatory possibilities and role of other mechanisms that can be put forth and tested. Hence, a major limitation of our evaluation is the number of such rival explanatory theories that can be practically put to test. While acknowledging this practical limitation, it may be said that a critical mass of realist evaluations will strengthen the explanatory power of the middle-range theories tested by these evaluations [15].

## Endnotes

^a^Critical realism is a philosophical position in social sciences that approaches causation within the social realm as being possible through rationally choosing from rival theories, thus advancing the ‘explanatory power’ of theories. According to Pratschke (2003), in critical realism, “*the ‘black-box’ of causation could be approached by understanding the gaps in the ‘generative mechanisms’ which may subsequently be explained by positing the existence of additional mechanisms at a deeper or more fundamental level*” [57].

^b^Block programme managers (BPM) are a new cadre of health managers created under the National Rural Health Mission (NRHM). These are young and typically recent graduates from management courses. BPMs operate at the *taluka* level. Similar cadres of non-medical health managers were created at the district and state levels as well.

^c^This was calculated by computing an average of percentage utilization rates of budgets of all facilities in the *taluka*/district.

^d^The National Rural Health Mission (NRHM) is a flagship programme of the Indian government to strengthen government health services through greater financial allocation and human resources. Under the NRHM, there was an induction of new cadres of health workers and health managers from village level upwards to PHC, *taluka*, district, and state levels. Decentralised planning and increased participation of elected representatives in formal structures within health services were key features of NRHM.

^e^PIP stands for programme implementation plan. The PIP is the annual action plan instituted by the NRHM. As per the NRHM, the PIP is an instrument for decentralised planning.

^f^ZP stands for *Zilla Panchayat*, the local governments at the district level.

## Electronic supplementary material

Additional file 1: **Abstract in Kannada.** (PDF 151 KB)

Additional file 2: **Abstract in Hindi.** (PDF 134 KB)

## Abbreviations

AMO: Administrative medical officer
CMO: Context mechanism outcome
CS: Caesarean section
NRHM: National rural health mission
PHC: Primary health centre
PRI: Panchayati Raj Institutions
THO: *Taluka* health officer.
