# Supplementary material for: Advancing the application of systems thinking in health: a realist evaluation of a capacity building programme for district managers in Tumkur, India
Source: Health Res Policy Syst. 2014 Aug 26;12:42. doi: 10.1186/1478-4505-12-42 (PMC4245764; doi:10.1186/1478-4505-12-42)
Supplement: Supplementary file 1 — Additional file 1: Abstract in Kannada. (PDF 151 KB) [file 12961_2013_343_MOESM1_ESM.pdf]

ಆರೋಗ್ಯ ವ್ಯವಸ್ಥೆಗಳ ಚಿಂತನೆಯ ಅನ್ವಯಿಸುವಿಕೆ ಮುಂದುವರಿಸುವುದು -ಜಿಲ್ಲೆಯ ವ್ಯವಸ್ಥಾಪಕರಿಗೊಂದು ಸಾಮರ್ಥ ನಿರ್ಮಾಣ ಕಾರ್ಯಕ್ರಮದ ನೈಜ ಮೌಲ್ಯಮಾಪನ

ಮುನ್ನುಡಿ

ಆರೋಗ್ಯ ಸಂಸ್ಥೆಗಳ ಕಾರ್ಯನಿರ್ವಹಣೆಯ ಸುಧಾರಣೆಗಾಗಿ, ಸಾಮರ್ಥ್ಯ ನಿರ್ಮಾಣದಂತಹ ಆರೋಗ್ಯ ವ್ಯವಸ್ಥೆಯ ಮದ್ಯಸ್ತಿಕೆಯನ್ನು, ಸಾಮಾನ್ಯವಾಗಿ ಜಿಲ್ಲೆಗಳಾದ್ಯಂತ ಅಳವಡಿಸಲಾಗುತ್ತದೆ
ಆಧರೆ, ಇಂತಹ ಮದ್ಯಸ್ತಿಕೆಗಳು, ಕೆಲವು ಆರೋಗ್ಯ ಕೇಂದ್ರಗಳಲ್ಲಿ ಅಥವಾ ಉಪ ಜಿಲ್ಲಾ ವ್ಯವಸ್ಥೆಗಳಲ್ಲಿ ಮಾತ್ರ ಪ್ರಯೋಜನಕಾರಿಯಾಗಿ, ಇತರೆಡೆಗಳಲ್ಲಿ ಕಾರ್ಯನಿರ್ವಹಿಸುವುದರಲ್ಲಿ ಫಲಕಾರಿಯಾಗುವುದಿಲ್ಲ. ಜಿಲ್ಲೆಯ ತಾಲುಕಾ ಕೇಂದ್ರಗಳನ್ನು (ಉಪ-ಘಟಗಗಳು) ಸಂಕೀರ್ಣ ಹೊಂದಾಣಿಕೆಯ ವ್ಯವಸ್ಥೆಯಾಗಿ ದೃಶ್ಯಿಕರಿಸಬಹುದು. ಇಂಥಹ ಕೇಂದ್ರಗಳು, ಸಾಮರ್ಥ್ಯ ನಿರ್ಮಾಣ ಮದ್ಯಸ್ತಿಕೆಗಳ ಒಳಹರಿವಿಗೆ ವಿವಿದ ರೀತಿಯಲ್ಲಿ ಪ್ರತಿಕ್ರಿಯಿಸುತ್ತವೆ. ಈ ಪ್ರತಿಕ್ರೀಯೆಯು, ಸ್ಥಳೀಯ ಪರಿಸ್ಥಿತಿಗಳು ಹಾಗು ಹಲವಾರು ಪ್ರತ್ಯೇಕ ಸಾಂಸ್ಥಿಕ ಮತ್ತು ಪರಿಸರ ಅಂಶಗಳನ್ನು ಅವಲಂಬಿಸಿರುತ್ತವೆ. ವಾಸ್ತಿಕ ಮೌಲ್ಯಮಾಪನವು, ಸಂಕೀರ್ಣ ಸ್ವಭಾವದ ಬದಲಾವಣೆಯನ್ನು ಜಿಲ್ಲಾ ಮಟ್ಟದ ಸಾಮರ್ಥವರ್ದನೆಯ ಹಸ್ತಕ್ಷೇಪವನ್ನು ಅರ್ಥಮಾಡಿಕೊಳ್ಳಲು ಅನುಕೂಲ ಮಾಡಿಕೊಡುತ್ತದೆ. ಸಂಧರ್ಭ ಅವಲಂಬಿತ ಯಾಂತ್ರಿಕ ಫಲಿತಾಂಶ, ಸಂರಚನೆಯನ್ನು, ಒಂದು ಸಲಕರಣೆಯಾಗಿ ಬಳಸಿದಾಗ ಆಗುವ ವಾಸ್ತವಿಕ ವಿಶ್ಲೇಷಣೆಯನ್ನು ನಾವು ಒಂದು ಗುರಿಯಾಗಿ ಪ್ರದರ್ಶಿಸುತ್ತೇವೆ. ಈ ವಿಶ್ಲೇಷಣೆಯು, ಪಲಿತಾಂಶಗಳು ಏಕೆ ಮತ್ತು ಹೇಗೆ ಸಂಭವಿಸಿವೆ ಎಂಬುದನ್ನು ಸಂಧರ್ಬ ಹಾಗೂ ಯಾಂತ್ರಿಕ ಬದಲಾವಣೆಯನ್ನು ಪರಿಗಣಿಸಿ ವಿವರಿಸುತ್ತದೆ.

ವಿಧಾನಗಳು

ಈ ಸಂಸ್ಲೇಷನೆಯನ್ನು ಎರಡು ತಾಲೂಕುಗಳ ಪಲಿತಾಂಶಗಳನ್ನು ಹೊಲಿಸುವದರ ಮೂಲಕ ವಿವರಿಸುತ್ತೇವೆ, ಹಾಗೂ, ವಿವಿಧ ವ್ಯಗ್ತಿಗತ, ಸಂಸ್ಥಾನಿಕ, ಸೂಕ್ಸ್ಮ ಮತ್ತು ಸ್ಥೂಲ ಸಂಧರ್ಭೋಚಿತ ಅಂಶಗಳ ಪರಸ್ಪರ ಕ್ರಿಯೆಯಿಂದ ಉಂಟಾಗುವ ವಿವಿಧ ಪಲಿತಾಂಶಗಳ ಅನ್ವೇಷಣೆಯನ್ನು, ಗುಣಾತ್ಮಕ ಅಂಶಗಳು (ಸಂಧರ್ಶನ ಟಿಪ್ಪಣಿ ಹಾಗೂ ವೀಕ್ಷಣೆಯ ಟಿಪ್ಪಣಿ) ಹಾಗೂ ಪರಿಮಾಣ ಸಂಭಂಧಿ ಅಳತೆಯ ಸ್ವಯಂ ಬದ್ದತೆ ಹಾಗೂ ಮೇಲ್ವಿಚಾರಣೆಯ ಶೈಲಿಯನ್ನು ಆದರಸಿ ಮಾಡುತ್ತೇವೆ.

ಪಲಿತಾಂಶಗಳು

ವಿವಿಧ, ವ್ಯಗ್ತಿಗತ, ಸಂಸ್ಥಾನಿಕ ಹಾಗೂ ಪರಿಸರದ ಅಂಶಗಳ ಆಧಾರದ ಮೇಲೆ, ತುಮಕೂರು ತಾಲೂಕಗಳು ಮದ್ಯಸ್ಥಿಕೆಗಳಿಗೆ ವಿವಿಧ ರೂಪದಲ್ಲಿ ಪ್ರತಿಕ್ರಯಿಸಿದವು. ಕಾರ್ಯಬದ್ದ ಹಾಗೂ ಬದಲಾವಣೆ ತರಲು ಧನಾತ್ಮಕ ಗಾಡತೆ ಹೊಂದಿದ ಸಿಭಂದಿ ಇರುವ ತಾಲುಕದಲ್ಲಿ, ಮದ್ಯಸ್ಥಿಕೆಯು, ಅಸ್ತಿತ್ವದಲ್ಲಿರುವ ಅವಕಾಶಗಳೊಂದಿಗೆ ಒಂದುಗೂದುವುದರ ಮೂಲಕ ಕೆಲಸ ಮಾಡಿತು. ವಿಕೆಂದ್ರಿಕರಣ ಪ್ರಕ್ರಿಯೆಯ ಮೂಲಕ ಕಾರ್ಯ ಸುಧಾರಣೆಯಾಯಿತು. ಆದಾಗ್ಯೂ, ಸಂಸ್ಥೆಯ ಭದ್ದತೆಯು, ನಿರ್ಣಾಯಕವಾಗಲಿ ಅಥವಾ ಸಾಕಷ್ಟು ವಿಮೊಚನಕಾರಿಯಾಗಿರಲಿಲ್ಲ. ಇತರ ಎರಡು ತಲುಕಾಗಳಲ್ಲಿ, ಕಾರ್ಯಬದ್ಧ ಸಿಬ್ಬಂದಿಯು, ತಮ್ಮ ಅನಿಸಿಕೆಗಳನ್ನು ಕಾರ್ಯರೂಪ ತರುವಲ್ಲಿ ವಿಫಲರಾದರು. ಹಾಗೂ, ಸಂಸ್ಥೆಯ ಸಾಧನೆಯನ್ನು ಉತ್ತಮಪಡಿಸಲಾಗಲಿಲ್ಲ. ಆದರೆ,ಇನ್ನೊದು ತಾಲುಕದಲ್ಲಿ, ನಾಯಕತ್ವವು, ಭದ್ಧತೆಯ ಕೊರತೆಯನ್ನು ಸರಿದೂಗಿಸುವಲ್ಲಿ ಫಲಕಾರಿಯಾಯಿತು.ಈ ಕಾರ್ಯವನ್ನು, ಆಸ್ಪತ್ರೆಯ ತುರ್ತು ಪ್ರಸೂತಿ ರಕ್ಷಣೆ ಕಾರ್ಯವನ್ನು ಶಕ್ತಿಗತಗೊಳಿಸುವುದರ ಮೂಲಕ, ಮಾಡಲಾಯಿತು.

ಮುಕ್ತಾಯ

ಸ್ಥಳೀಯ ಆರೋಗ್ಯ ವ್ಯವಸ್ಥೆಯ ಸಾಮರ್ಥ್ಯ ನಿರ್ಮಾಣ ಕಾರ್ಯಕ್ರಮವನ್ನು ಸಂಸ್ಥೆಯ ಆಂತರಿಕ (ವ್ಯಗ್ತಿಗತ, ಸಂಸ್ಥಾನಿಕ) ಮತ್ತು, ಬಾಹ್ಯ (ಸಾಮಾಜಿಕ -ರಾಜಕೀಯ), ಲಕ್ಷಣಗಳ ನಡುವೆ ಇರುವ ಸಂಬಂಧಗಳನ್ನು ಒಂದುಗೂಡಿಸುವ ಅಥವಾ ಎದಿರಿಸುವ ಮೂಲಕ ಸಾದಿಸಬಹುದು. ಇಂಥಹ ಮದ್ಯಸ್ತಿಕೆಯ ವಿನ್ಯಾಸ ಹಾಗೂ ಅನುಷ್ಠಾನಕ್ಕೆ ಪ್ರಚೋದಕ ಹೊಂದಾಣಿಕೆಯ ಅವಕಾಶಗಳನ್ನು ಗುರುತಿಸುವ

ಅಗತ್ಯವಿದೆ. ಸ್ಥಳೀಯ ಆರೋಗ್ಯ ವ್ಯವಸ್ಥೆಗಳ ಉಪ-ಘಟಗಳ ಆಂತರಿಕ ಸಂರಚನೆ ಭಿನ್ನವಾಗಿರುತ್ತದೆ. ಹೀಗಾಗಿ, ಸಾಮರ್ಥ್ಯ ನಿರ್ಮಾಣ ಕಾರ್ಯಕ್ರಮವು ವಿವಿದ ಮಾರ್ಗಗಳ ಬದಲಾವಣೆ ಸಾಧ್ಯತೆಗಳಿಗೆ ಸ್ಥಳಾವಕಾಶ ಮಾಡಿ ಕೊಡಬೇಕಾಗುತ್ತದೆ.

ರಚಿಸುವ ಹಾಗೂ ಉಹಾಪರೀಕ್ಷೆಯ ಪ್ರಕ್ರಿಯೆಯ ಮೂಲಕ, ವಿಮರ್ಶಾತ್ಮಕ ಹೋಲಿಕೆಯ ಮೂಲಕ, ಪ್ರಾಯೋಗಿಕ ಮಾದರಿಗಳನ್ನು ಪತ್ತೆಹಚ್ಚುವ ಹಾಗೂ, ವ್ಯಾಪ್ತಿ ಮತ್ತು ವಿಸ್ತಾರಗಳ ಮೇಲ್ವಿಚಾರಣೆಯ ಮೂಲಕ, ಮಾಡಿದ ಒಂದು ವಾಸ್ತವವಾದಿ ಮೌಲ್ಯಮಾಪನವು, ಆರೋಗ್ಯ ವ್ಯವಸ್ಥೆಯ ಬದಲಾವಣೆಯ ಸಮಗ್ರ ಮೌಲ್ಯಮಾಪನಕ್ಕೆ ಅನುವು ಮಾಡಿಕೊಡುತ್ತದೆ.

ಪ್ರಮುಖ ಪದಗಳು:
ಸಾಮರ್ಥ್ಯ ನಿರ್ಮಾಣ, ವಾಸ್ತವಿಕ, ಮೌಲ್ಯಮಾಪನ, ಕಾರ್ಯಕ್ರಮ ಸಿದ್ಧಾಂತ, ಜಿಲ್ಲಾ ಆರೋಗ್ಯ ವ್ಯವಸ್ಥೆ, ವ್ಯವಸ್ಥೆಯ ಚಿಂತನೆ, ಸಂಸ್ಥಾನಿಕ ಬದ್ಧತೆ, ಸ್ವಯಂ- ಪರಿಣಮಕಾರಿತ್ವ.
