# Supplementary material for: Advancing the application of systems thinking in health: a realist evaluation of a capacity building programme for district managers in Tumkur, India
Source: Health Res Policy Syst. 2014 Aug 26;12:42. doi: 10.1186/1478-4505-12-42 (PMC4245764; doi:10.1186/1478-4505-12-42)
Supplement: Supplementary file 2 — Additional file 2: Abstract in Hindi. (PDF 134 KB) [file 12961_2013_343_MOESM2_ESM.pdf]

स्वास्थ्य में सोच प्रणालियों के आवेदन में आगे बढना: तुमकुर ज़िला, भारत में ज़िला प्रबंधकों के लिए एक क्षमता निर्माण कार्यक्रम का एक यथार्थवादी मूल्यांकन (रियलिस्ट इवैल्यूएशन)

परिचय

स्वास्थ्य केंद्रों की उपयोगिता में वृद्धि लाने के दृष्टिकोण को सामने रखते हुए ज़िला स्तरों पर वर्तमान स्वास्थ्य प्रबंधन पद्धतियों में कई बार बदलाव लाने के प्रयत्न किए गये हैं| इनके बावजूद भी ये बदलाव ज़िला स्तरों के सभी स्वास्थ्य केंद्रों एवं अस्पतालों में उपयोगी साबित न होते हुए, केवल कुछ ही केंद्रों में उपयोगी साबित हुए हैं| इन बदलाव की उपयोगिता इन केन्द्रों के अलग-अलग पद्धतियों पर निर्भर हैं| क्षमता संवर्धन कार्यों की अलग-अलग प्रकार की ज़िम्मेदारियाँ हैं, जो इन केन्द्रों के कुछ विशिष्ट व्यक्तियों, संस्थाओं, स्थानीय एवं नैसर्गिक परिस्थितियों और वहाँ के वातावरण पर आधारित होती हैं| यथार्थवादी मूल्यांकन (रियलिस्ट इवैल्यूएशन) दृष्टिकोण द्वारा दक्षिणी भारत के तुमकुर ज़िले में स्थानीय स्वास्थ्य प्रणालियों के भीतर संगठनात्मक परिवर्तन को समझने का प्रयास का यहां वर्णन किया गया है| कठिन परिस्थितियों में भी ज़िला या तालुकों के अलग-अलग स्तरों पर क्षमता संवर्धन कैसे काम करता है, इसको समझने से क्षमता संवर्धन कार्यक्रमों में सुधार लाया जा सकता है | यथार्थवादी मूल्यांकन दृष्टिकोण स्वास्थ्य देखभाल स्थितियों में जटिल प्रणालियों के उपगोग के बारे में हमारी समझ में सुधार लाता है।

विधियां

हम तुमकुर ज़िले के तालुकों के प्रदर्शन के कई निवेश, प्रक्रिया और परिणाम मात्रों पर डेटा एकत्र की। उसके बाद हमने तालुकों के प्रदर्शन में योगदान करने में विभिन्न व्यक्तिगत, संस्थागत, और प्रासंगिक कारकों के परस्पर प्रभाव को पता लगाने का प्रयत्न किया । हमने गुणात्मक डेटा (इंटरव्यू टेप और प्रेक्षण नोट्स) और स्वस्थ्य प्रबंधकों के प्रतिबद्धता, आत्म प्रभावकारिता और पर्यवेक्षण शैली की मात्रात्मक मोल का इस्तेमाल किया।

परिणाम

तुमकुर ज़िले के तालुकों ने क्षमता निर्माण पहल की वजह से अलग प्रतिक्रिया व्यक्त कीं। यह प्रतिक्रिया कई व्यक्तिगत, संस्थागत और पर्यावरणीय कारकों के बीच पारस्परिक प्रभाव से समझाया जा सकता है। प्रतिबद्ध कर्मचारियों और परिवर्तन करने के लिए सकारात्मक इरादों के मौजूदी में, एक तालुका में क्षमता संवर्धन कार्य्रकम ने, तालुका में उपलब्ध मौजूदा अवसरों के साथ मिलके परिणाम लाया। यह अवसर भारत के कई राज्यों में चल रही विकेन्द्रीकरण प्रक्रिया से उपलभ्ध हुआ। हालांकि, संगठन के प्रति प्रतिबद्धता महत्वपूर्ण है किन्तु सिर्फ प्रतिबद्धता पर्याप्त न था। दो अन्य तालुकों में प्रतिबद्ध कर्मचारियों संगठन के प्रदर्शन में सुधार करने के अपने इरादों को साकार करने में असमर्थ थे। जबकि एक और तालुका में, नेतृत्व ने प्रतिबद्धता की कमी की भरपाई करने में सक्षम रहा।

समापन

स्वास्थ्य प्रणाली की क्षमता निर्माण कार्य्रकम, स्वास्थ्य केंद्रों की आंतरिक (व्यक्तिगत और संगठनात्मक) और बाह्य (नीति और सामाजिक राजनीतिक वातावरण) के बीच मौजूदा संबंधों के मिलाव या मुकाबले के माध्यम से काम कर

सकते हैं। इस तरह के क्षमता निर्माण कार्यक्रम के रचना और कार्यान्वियन में ऐसे ट्रिगर संरेखण के लिए अवसरों की पहचान करने की आवश्यकता है। स्थानीय स्वास्थ्य प्रणालियां अपने आंतरिक विन्यास में अलग हो सकते हैं, इसलिए क्षमता निर्माण कार्यक्रमों को अलग-अलग रास्ते के माध्यम से बदलाव के संभावनाओं को समायोजित करने की आवश्यकता है।

एक यथार्थवादी मूल्यांकन (रियलिस्ट इवैल्यूएशन) में, हम परिकल्पना का परीक्षण करके, आलोचनात्मक तुलना कर, अनुभवजन्य पैटर्न की खोज और उनके गुंजाइश और परिवर्तन की सीमा की निगरानी के द्वारा, स्वास्थ्य प्रणाली में परिवर्तन का एक व्यापक मूल्यांकन कर सकते हैं।

यह सार कन्नड़ और अंग्रेजी भाषाओं में भी उपलब्ध है।
